# Revised Protein Sparing Diet in Obesity and Type 2 Diabetes Mellitus

**DOI:** 10.3390/nu14245325

**Published:** 2022-12-15

**Authors:** Raffaele Ivan Cincione, Francesca Losavio, Giuseppe Cibelli, Giovanni Messina, Rita Polito, Elias Casula, Pamela Pia Cincione, Marco Amatruda, Pierpaolo Limone

**Affiliations:** 1 Department of Clinical and Experimental Medicine, University of Foggia, 71122 Foggia, Italy; 2 IRCCS Santa Lucia Foundation, 00128 Rome, Italy; 3 Faculty of Medicine, University of Foggia, 71122 Foggia, Italy; 4 Department of Humanities, University of Foggia, 71122 Foggia, Italy

**Keywords:** revised protein sparing diet, type 2 diabetes mellitus, obesity, body composition, indirect calorimetry, free fat mass, fat mass, ectopic fat, metabolic inflexibility, diabetes reversal

## Abstract

Effective nutrition therapy is a pressing issue in obesity and type 2 diabetes mellitus (T2DM) management. As such, this research aimed to determine the performance of a revised dietary strategy built on the protein-sparing diet in obesity and type 2 diabetes mellitus with regard to obtaining a rapid and stable improvement in glucometabolic control, body weight, body composition, and energy metabolism when applying the strategy in just twenty-one days. The revised protein-sparing diet differs from the traditional protein-sparing modified fast (PSMF) because it does not include foods. The daily calorie intake of this diet is exclusively derived from Isolate whey protein in addition to a formulation of Isolate whey protein enriched with essential amino acids in free form, with the addition of lipids such as extra virgin olive oil and coconut oil as a source of medium chain fatty acids, where the latter is taken for only the first four days of the diet, together with the use, for the same duration, of extended-release metformin, as the only antihyperglycemic allowed. Anthropometric measurements, bioimpedance analysis, indirect calorimetry, and blood chemistry assessments were conducted at the beginning of the study, time 0 (T0), and at the end, time 1 (T1), i.e., on the 21st day. The main outcomes of the revised protein-sparing diet after only twenty-one days were a reduction in body weight with the predominant loss of visceral atherogenic abdominal fat and, therefore, a possible contextual reduction in ectopic fat deposits together with a simultaneous reduction in insulin resistance and normalization of insulin levels, maintenance of free fat mass and basal metabolism, restoration of metabolic flexibility, and improvement of the glucometabolic and lipidic parameters. These results demonstrate the promising potential of the revised protein-sparing diet as an “etiologic tool” in the integrated nutritional treatment of metabolic diseases such as obesity and type 2 diabetes mellitus.

## 1. Introduction

Metabolic diseases such as obesity and type 2 diabetes mellitus (T2DM) are considered 21st-century global epidemics that can affect people of all ages, with their incidence and prevalence continuing to dramatically increase. These two conditions determine increased morbidity and mortality derived from their associated chronic comorbidities, such as cardiovascular diseases, which lead to reduced life expectancy and high costs for the health system [1,2,3,4].

For this purpose, a healthy lifestyle and body weight are essential steps in the management of both pathologies and related complications. Current therapeutic approaches include lifestyle modification, pharmacologic therapy, and bariatric surgery. Regarding lifestyle interventions, such as the Mediterranean diet that exert positive health effects of the high content of nutraceutical components of food used [5,6,7,8], calorie reduction is always the decisive element for achieving weight loss, with the fundamental need to reduce fat mass while preserving lean mass. However, low-calorie diets, have a high dropout rate and considerable heterogeneity in outcomes [9,10,11,12].In this regard, the protein-sparing approach was developed by Blackburn and Bistrian [13] in their early work in 1970, thanks to pioneering studies of the causal link between protein–calorie malnutrition due to protein hypercatabolism, resulting in increased mortality in hospitalized patients. Blackburn and his collaborators thus laid the theoretical foundations of protein-saving treatment by using high biological value proteins to antagonize protein catabolism and, at the same time, preserve lean body mass and the basal metabolic rate. Protein sparing has therefore become the conceptual framework underlying the protein-sparing modified fast, as we know it today, for treating obesity [14,15] and other diseases [16,17,18,19,20,21,22,23]: a dietary system, more precisely, a form of controlled starvation and ketosis, with very limited caloric intake, usually <400 kcal/day, which includes only a proteins intake in the amount of 1.2–1.5 g per kg of ideal body weight, and a carbohydrate intake <10 g within 24 h with a minimum quota of fats in the quantity of 10 g per day to reduce the occurrence of cholelithiasis, and a large intake of water (two liters) together with vitamin and mineral supplement to compensate for the obvious nutritional deficiencies in this dietary approach.

Within two or three days from the start of this treatment, with a near-zero carbohydrate intake, a shift takes place in the energetic substrate selection, with the predominant oxidation of triglycerides and consequent hepatic production of ketone bodies, which act as energy molecules, such that protein intake covers the anticatabolic and plastic muscle requirements. The body fat reduction is approximately 0.2 kg per day for females and 0.3 kg per day for males, and it has been confirmed as a safe, rapid weight reduction method. Moreover, the positive effects of the ketosis induced by the protein-sparing modified fast also include endocrine, metabolic, and cardiovascular effects, with significant improvement of the glycemic and lipidic profile and blood pressure values [16,24]. Therefore, this dietary approach, rigorously medically monitored, provides outstanding results for rapid and safe weight reduction and glucometabolic control, even over the long term and, also recently, in the management of childhood overweight and obesity [25,26]

Considering this evidence, the present research aimed to test a revised nutritional approach developed from the protein-sparing modified fast model used for just twenty-one days in obese patients with T2DM, relative to the following clinical outcomes: assess the safety of the nutritional protocol and the compliance of patients together with the improvement of the glucometabolic control, body weight, body composition, energy metabolism, metabolic flexibility and possibly in the reduction of ectopic fat. The revised protein-sparing diet differs from the original PSMF protocol because it does not include foods. The daily calorie intake is exclusively derived from Isolate whey protein in addition to a formulation of Isolate whey protein enriched with essential amino acids in free form, with the addition of lipids in the form of extra olive virgin oil and coconut oil’s medium chain fatty acids, where the latter is taken for only the first four days of the diet. The revised protein-sparing diet also includes, for the same duration, the use of extended-release metformin as the only antihyperglycemic drug. The protein load prevents muscle catabolism, whereas the state of ketosis obtained by eliminating all dietary carbohydrates and protein intake help to suppress appetite, which consequently increases compliance with the nutritional regime.

## 2. Patients and Methods

### 2.1. Patients

At the clinical section of Diet therapy and Metabolic Diseases of the University of Foggia, fifty obese diabetic patients (25 female and 25 male; average age: 47 ± 10 years; BMI: 33.9 ± 5.2 kg/m^2^; HA1c: 7.98 ± 0.17) were recruited, with the following inclusion criteria: age 18–65 years, body mass index >30 kg/m^2^, normal renal function, normal hepatic function, obesity, and T2DM not treated with insulin. Exclusion criteria included pregnancy or breastfeeding, hyperuricemia or gout, neoplastic disease, corticosteroid, chronic inflammatory therapy, the presence of diseases other than obesity, and T2DM. After initiating the experimental protocol, all subjects suspended the use of antihyperglycemic drugs, as per Steven et al. [27] with the exception of metformin, as extended-release metformin. The enrolled patients were asked not to change any other ongoing therapies, and hypertensive medications were either lowered or discontinued depending on the patient’s clinical need. The enrolled patients signed a written authorization. The research protocol was approved by the ethical committee (Protocol: CT22_2018).

### 2.2. Clinical Experimental Design

The study is a single-center prospective longitudinal single-arm dietary pilot trial and no sample size was calculated.

**Figure 1:**
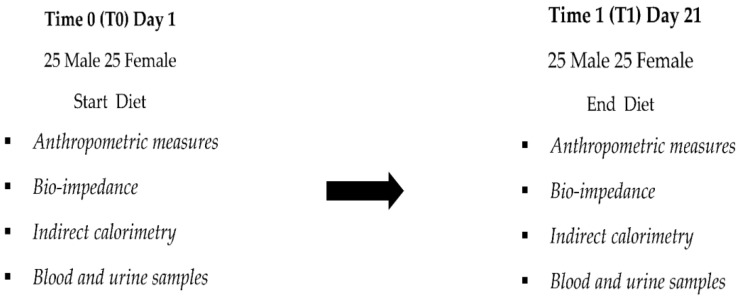
Study design.

Based on the mean difference in HbA1c (%) and SD in prior research [28], 15 participants per group are needed to identify a potential treatment effect with a 5% significance value and 80% study power. Anthropometric measurements, bioimpedance analysis, indirect calorimetry, and blood chemistry assessments were conducted at the beginning of the study, time 0 (T0), and at the end, time 1 (T1), i.e., on the 21st day. The clinical study design is reported in Figure 1.

### 2.3. Anthropometry

#### Indirect Calorimetry Measures

Height, weight, body mass index, waist circumference, hip circumference, and waist-to-hip ratio, were measured [27].

### 2.4. Body Composition

The body composition of the patients was assessed through a multi-segmental multi-frequency bioelectrical impedance analysis with an eight-polar tactile electrode placed on each palm, thumb, and sole. The eight-polar tactile-electrode assessed arm, trunk, and leg impedance at 1, 5, 50, 250, 500, and 1000 kHz, (InBody 770. Co., Ltd., 625 Eonju-Ro, Gangnam-gu, Seoul, Republic of Korea) [29,30,31], to evaluate fat mass (FM), Kg; fat-free mass (FFM), Kg; muscle mass (MM), kg; total body water (TBW), L; visceral fat area (VFA), cm^2^ [32,33,34,35].

### 2.5. Indirect Calorimetry

All the patients were assessed via indirect calorimetry using the Vyntus CPX Canopy Metabolic Cart with SentrySuite Software (Vyaire Medical Carefusion, 97204 Höchberg, Germany) to evaluate O_2_ and CO_2_ volumes, and the respiratory quotient (RQ). (O_2_ electrochemical analyzer, accuracy: 0.05 vol%, resolution: 0.01% O_2_, CO_2_ analyzer thermal conductivity, accuracy: 0.05 vol%, resolution: 0.005% CO_2_). We used the following gas mixture: 12.0% oxygen, 5% carbon monoxide, and N2 to maintain the balance between the various gaseous components. The levels of VO_2_ and VCO_2_ were measured after a steady state condition when there had been no variation exceeding 5%. REE was determined according to the Weir equation without the use of urea nitrogen levels in urine [36].

### 2.6. Biochemical Analysis

The hepatic enzyme, glycemia, glycosylated hemoglobin, total cholesterol, high-density lipoprotein cholesterol, low-density lipoprotein cholesterol, triacylglycerol, insulinemia, and uric acid were analyzed at the Clinical Pathology laboratory of the Policlinico Riuniti of Foggia. Each patient has measured ß-hydroxybutyrate level on capillary blood on a day-to-day basis at home (GlucoMen AREO 2K ß-Ketone Sensor, EN ISO 15197:2015, Menarini Diagnostics, Florence, Italy). The HOMA index was computed to assess insulin resistance. [37].

### 2.7. The Revised Protein Sparing Diet Experimental Protocol: Dietary, Pharmacological, and Supplementation Program

The protein powder needs of each patient were determined considering a quantity of 1.2 ± 0.3 g of proteins not for each kg of ideal body weight but for each kg of free fat mass, estimated through bioimpedance analysis, at study start [27] with an average daily protein value between 60 and 80 g. The protein intake was provided partly as whey protein Isolate powder (Star Whey, Named, Lesmo, Milan, Italy) and partly as two bags of whey protein Isolate enriched with free essential acids (Aminexem, Named, Lesmo, Milan, Italy). Patients could decide, at their discretion, how much water to use to dissolve the protein powder and split it up into five portions during the day. The Isolate milk whey protein powder and the isolate milk whey protein powder enriched with free essential acids had a high protein biological value with BV = 104 [38] and high digestibility due to the presence of proteolytic enzymes in both protein preparations, and near-to-zero carbohydrates, fats, and lactose. The average nutritional values per 100 g of the Isolate milk whey protein powder were 90 g of protein, 1.40 g of carbohydrates, 1.69 g of fats, 10 mg of protease blend with standardized enzymatic activity 350 min HUT/mg, hemoglobin unit tyrosine base, 2.8 mg of vitamin B6 (pyridoxine or hydrochloride), 2.8 mg of vitamin B2 (riboflavin), 2.2 mg of vitamin B1 (thiamine hydrochloride), for 381 total/kcal. The average nutritional values for two bags of the Isolate milk whey protein powder enriched with a specific mixture of essential free amino acids were protein powder 31 g, carbohydrates 1.2 g, fats 0.4 g, essential free amino acid 17.8 g equivalent to 57% EAA value (where the highest content in EAA found in nature has an EAA of 46–47%), isoleucine 2.69 g, phenylalanine 0.69 g, leucine 4.08 g, lysine 3.58 g, methionine 0.50 g, threonine 1.52 g, tryptophan 0.32 g, histidine 1.81 g, leucine isoleucine 4.08 g, isoleucine 2.69 g, valine isoleucine 2.58 g, vitamin D 50, thiamine 1.12 mg, riboflavin 1.4 mg, vitamin B6 1.4 mg, and plant protease, papain 250 mg with enzymatic activity 50.000 TU, tyrosine unit/mg/min, bromelain 250 with enzymatic activity 250 GDU, gelatin digestion unit, with a total of 133 kcal for two bags. The diet in this study did not include foods. 10 g of extra virgin olive oil represent the daily amount of lipids. For the initial four days of the nutritional treatment, patients also received 10 milliliters of pure medium chain triglycerides (MCTs) from coconut oil. The MCTs were in the form of C8 caprylic acid and C10 capric acid (Ketofast, Named Italia, Lesmo, Milan, Italy). For the same duration, patients also received extended-release metformin, with an average daily dosage between 500 and 1500 mg.

A daily intake of 25 mg of fiber was provided as a supplement (Psyollogel, Nathura, Giuliani Pharma, Milan, Italy).

The dietary protocol lasted twenty-one days. The average daily caloric content was approximately 400 kcal/day. Unsweetened nonalcoholic drinks, such as coffee, herbal tea, tea, and infusions, were allowed. Because micronutrients were not balanced in the treatment, one daily caplet of multimineral multivitamins (Multicentrum, GlaxoSmithKline S.p.A., Verona, Italy) was integrated into the nutritional intervention to avoid deficiencies [39] In addition, every patient was instructed to drink a minimum of two liters of water daily as part of the treatment plan and to take all the supplements, including potassium and magnesium, amounting to six tablets per day, giving a total of 300 mg of potassium and 113 mg of magnesium (Polase, GlaxoSmithKline S.p.A., Verona, Italy). Following the completion of the revised protein-sparing diet, each patient undertook a slow and gradual reintroduction of carbohydrates and lipids over a period of 8–12 weeks.

### 2.8. Statistics

To verify the normality of distribution, data were first analyzed with a Shapiro-Wilks test. After the analysis was performed, before (pre-diet, baseline T 0 day) and after the diet (post-diet, T 21 day), by means of paired t-tests or sign-rank Wilcoxon test, depending on values distribution. To prevent Type I errors, we used the Bonferroni correction. Significance was set at α = 0.05/15 = 0.003 The analyses were performed using SPSS version 22 (SPSS Inc., Chicago, IL, USA)

The whole set of data was also analyzed by calculating Δ% (i.e., delta percent) to estimate the time differences of each parameter between the start and the end of treatment, and assessed according to the following formula:

Δ% = [(Z − W)/W] × 100

where Δ% is the percentage difference between each parameter and the respective base value, expressed as the ratio of the exact deviation from the base value.

## 3. Results

Our patients have been fully compliant, with no dropouts. Non-parametrical sign-rank Wilcoxon analysis of pre and post-diet effects, as in Table 1 and Table 2, showed a significant reduction, with *p* < 0.001, for bw, body weight (kg),(z = −4.782); bmi, body mass index, (kg/m^2^),(z = −4.784); whr, waist-to-hip ratio (z = −3.682); wc, waist circumference (cm), (z = −4.798); hp, hip circumference (cm), (z = −4.789); fm, fat mass kg, (z = −4.783); vfa, visceral fat area (cm^2^),(z = −4.784); ffm, free fat mass (kg), (z = −4.084); mm, muscle mass (kg), (z = −4.125); tbw, total body water (l), (z = −4.618); bm, basal metabolism (kcal), (z = −4.587); rq, respiratory quotient, (z = −4.306). VO_2_ (mL/min), VCO_2_ (mL/min), VO_2_/Ffm (z = −1.067), and REE/Ffm (z = −1.689) showed a not significant reduction.

Non-parametrical sign-rank Wilcoxon analysis of pre and post-diet effects on blood values, showed a significant reduction, *p* < 0.001, for total cholesterol (mg/dL) (z = −4.783); ldl cholesterol (mg/dL) (z = −4.783); hdl cholesterol (mg/dL) (z = −2.786); insulin (μU/mL) (z = −4.752); triglycerides (mg/dL) (z = −4.228); glucose (mg/dL) (z = −4.784); hbA1c (%) (z = −4.803); homa-ir (z = −4.803); ketonemia (mmol/L) (z = −4.782). The following variable was not significantly different between the two-time points: uric acid (mg/dL) (z = −0.524), as in Table 3.

## 4. Discussion

Here, we demonstrated that the revised protein-sparing diet is an impactful dietary intervention safe and with a high level of compliance, which induces a rapid improvement in the patient’s glucometabolic control, body weight, body composition, energy metabolism, metabolic flexibility, and possibly in reducing ectopic fat in obese patients with T2DM over a period of twenty-one days.

We have obtained a significant reduction, on average by −8% ± 2 as Δ% in body weight and body mass index. Abdominal fat mass significantly decreases as attested by a decrease in the waist and hip circumferences, −6.32 and –5.27 cm and as Δ% −5.99% and −4.45% respectively, and more directly by the visceral fat area with a Δ% of −10.97% equal to 24.83 cm^2^, all included in the loss of fat mass equal to = −5.18 kg and a Δ% of −14.38% [40,41]. Therefore the revised protein-sparing diet, can reduce visceral fat in the abdominal region [42] and consequently decreases the cardiometabolic risk [43]. Furthermore, the change in the respiratory quotient, from an initial value of 0.94 to a value of 0.75 at the end of the diet, indicates a full shift in the selection of the oxidized energy substrate toward the elective use of lipids, finally leading to the synthesis of ketones [44,45].

As stated in guidelines for treating obese and diabetic patients, the rapid weight reduction achieved by low-calorie diets leads to a loss in lean muscle mass and performance [46]. Our results confirm those of other authors [47,48,49,50,51] in showing that is possible to preserve lean mass during a very low-calorie regime such as through the revised protein-sparing diet in obese subjects with type 2 diabetes mellitus. An initial decrease in lean mass was apparent, with an average variation of free fat mass of −2.40 kg and −2.19 kg of muscle mass, but the average variation of total body water was −2.05 kg. Thus, the decrease in free fat mass and muscle mass is almost entirely due to the decrease in total body water and to the use of bioimpedance analysis, The bioimpedance analysis estimates body water and, above all, the value of body impedance as parameters directly influenced by the state of body hydration. The reduction in total body water during the revised protein-sparing diet derived from increased excretion of water for the elimination of ketone bodies, reduction in dietary sodium intake, and depletion of liver and muscle glycogen [52,53]. We can confirm that the revised protein-sparing diet had an intense sparing action on lean body mass because of individualized protein intake and the presence of essential amino acids in free form, which exert highly intense anabolic action. In addition, the preserving action on lean body mass also allows a reduction in basal metabolism to be avoided. It is commonly acknowledged that extreme calorie restriction decreases basal metabolic rate [54,55,56]. As reported by other authors [54,55] our findings highlight a not statistically significant reduction in VO_2_/Ffm and Ree/Ffm ratios. Therefore, both the metabolic rate and the energy rate do not change with dietary intervention. This strengthens the hypothesis that the revised protein-sparing diet does not affect the basal energy expenditure, which is directly associated with the maintenance of the metabolically active mass, and improves metabolic flexibility as also attested by the respiratory quotient simultaneous decrease. The revised protein-sparing diet had important glucometabolic effects, with a significant reduction in the HOMA index with an average Δ% equal to = −85.4% and insulin, fasting blood glucose, and glycated hemoglobin with a Δ% of −36.07%, −23.10% and −12.04% respectively. This dietary intervention also results in an improved lipid profile with a significant reduction in total cholesterol Δ% −19.74%, LDL cholesterol Δ% −22.48%, and triglycerides Δ% −20.05%. The changes in blood HDL cholesterol and uric acid level between the start and end of treatment were not statistically significant. These findings are consistent with those of previous research. These results are consistent with those of other research. [50,51,57,58,59]. In all patients, none had renal adverse reactions during the revised PSMF and the administration of free amino acids, as in our investigation [60].

The most innovative and original features of the revised protein-sparing diet lie in the use of Isolate whey protein and Isolate whey protein enriched with essential amino acids in a free form together with the addition of lipids in the form of extra olive virgin oil and coconut oil as a source of medium chain fatty acids as the only daily calorie intake for twenty-one days. As already mentioned above the revised protein-sparing diet does not include foods.

The administration of medium-chain fatty acids from coconut oils is useful for various reasons. The specific timing of administration, i.e., only for the first four days of treatment, helps significantly in reducing the effects related to the patient’s keto-adaptation phase, a condition that can occur with a very restrictive nutritional diet such as the revised protein-sparing diet.

As demonstrated by Cahill [61,62], the activation of lipolysis is progressively established starting between 72–96 h after the beginning of the diet, and the production kinetics of blood ß-hydroxybutyrate show that it reaches its almost maximum concentration value on the twentieth day. Hence, the medium-chain fatty acids from coconut oils are administered for only 96 h, and the revised protein-sparing diet is undertaken for only 21 days.

Restoring metabolic flexibility is one of the more significant aspects of the revised protein-sparing diet, a keystone for weight reduction and, above all, for reversing diabetes [27,47,50,51,57]. Unlike long-chain fatty acids caprylic and capric acid enter the mitochondria, without needing carnitine as a transporter, and are rapidly converted to ketone bodies. In this way, we obtain a transient exogenous increase in ketonemia, pending the production of endogenous ketone bodies derived from the patient’s lipolysis of adipose tissue triglycerides. In addition, through the rapid production of ketone bodies, the administration of caprylic and capric acid contributes to reductions in both blood glucose levels and insulin resistance [63] together with the action of extended-release metformin, the only oral antidiabetic drug that was not discontinued during this research protocol. Caprylic acid, capric acid, and extended-release metformin are administered only for the first four days of the diet and then suspended until 21 days. This is a crucial element: insulin resistance and the resulting hyperinsulinemia exert an inhibitory action on lipolysis and, therefore, on the endogenous production of ketone bodies, thus preventing the onset of the ketosis condition. At the same time, the administration of medium-chain fatty acids, caprylic acid, and capric acid improves lipid oxidation, increasing energy consumption and thus promoting mitochondrial metabolic flexibility [63]. Furthermore, the revised PSMF also induced a change in the respiratory quotient value from the initial value of 0.94 to a value of at the end of treatment of 0.75, attesting to the prevalent use of lipids as an energetic substrate [44,64,65]. Therefore, all patients had a transition from a state of metabolic inflexibility to a state of metabolic adaptability. a key element that may contribute toward reducing especially the visceral adipose tissue accumulated in the liver, pancreas, and muscle in patients with obesity and type 2 diabetes mellitus. This nutritional approach allows rapid activation of the ketosis, with a maximum increase of 3.2 mmol/L in ß-hydroxybutyrate concentrations and an average value of 1.7 mmol/L, because of the complete absence of dietary carbohydrates. Under these conditions, lipolysis is intensely activated particularly in visceral fat. In this way, the revised protein-sparing diet may contribute to reducing cellular lipotoxicity to an individual lipidic cut-off value, and thus, as demonstrated, restoring normal cellular functions. All these results align with Taylor’s findings and the demonstrated mechanisms of personal fat threshold and the twin cycle as the conceptual and practical basis for diabetes reversal [27,50]. In other words, the recovery of metabolic flexibility allows us to characterize the revised protein-sparing diet as a kind of “metabolic-nutritional gym” that is able to exert a sudden reset, restoring the management of mitochondrial energy substrates and consequently contributing to a reduction in insulin resistance.

## 5. Conclusions

In this single-arm pilot study, we demonstrated the usefulness of the revised protein-sparing diet, based on the administration of Isolate whey protein and whey protein Isolate enriched with free essential amino acids together with the addition of caprylic and capric acids and metformin. This nutritional approach is a highly effective integrated clinical nutritional protocol able to directly antagonize the physiopathological processes in obese patients with type 2 diabetes mellitus. In fact, in just twenty-one days, we obtained reductions in body weight with the predominant loss of fat mass, possible reductions in ectopic fat deposits, maintenance of free fat mass and basal metabolism, and improvements in glucometabolic and lipidic parameters. The study limitations include the lack of follow-ups and lack of a control arm. Because of these limitations, the current results must be considered as a preliminary until further randomized case-control studies are conducted to confirm the findings obtained in this pilot study.

However, in our opinion, the results propose, reinforce and amplify the fundamental importance of preserving the lean mass during a very-low-calorie dietary regime, such as the revised protein-sparing diet in treating obese subjects with type 2 diabetes mellitus, together with a contextual intense and selective reduction in visceral and possibly in ectopic fat. These elements, within the general conceptual framework of improved body composition and restored metabolic flexibility, demonstrate the feasibility of stimulating a reversal of obesity and diabetic diseases through tailored nutritional intervention. In light of this preliminary evidence, the revised protein-sparing diet is proposed as an impact treatment not only in the management of any classes of obesity, also as a possible integrative treatment for bariatric surgery but especially for obese diabetic patients, where treatment can lead to a significant reduction in hypoglycemic therapy through the possible restoration of the function of the pancreatic beta cells and normal insulin sensitivity.

## Figures and Tables

**Table 1:** Characteristics of patients.

Number of patients	50
Men/Women	25/25
Age (years)	47 ± 10

**Table 2:** Anthropometric, body composition, and indirect calorimetry parameters before and after the revised protein-sparing diet.

Parameters Mean ± SE	Baseline T0 Mean ± SD	T1 Day 21 Mean ± SD	*p*-Value	Δ% T0–T21
Bw, Body Weight (kg)	94.74 ± 3.03	87.45 ± 2.96	*p* < 0.01 *	−7.83%
BMI, Body mass index (kg/m^2^)	33.98 ± 0.94	31.31 ± 0.91	*p* < 0.01 *	−7.96%
Whr, Waist-to-hip ratio	0.88 ± 0.01	0.86 ± 0.01	*p* < 0.01 *	−2.18%
Wc, Waist circumference (cm)	106.58 ± 2.47	100.26 ± 2.46	*p* < 0.01 *	−5.99%
Hc, Hip circumference (cm)	120.43 ± 2.34	115.16 ± 2.52	*p* < 0.01 *	−4.45%
Fm, Fat mass (kg)	38.75 ± 2.04	33.57 ± 2.07	*p* < 0.01 *	−14.38%
Vfa, Visceral fat area (cm^2^)	232.56 ± 6.62	207.73 ± 6.80	*p* < 0.01 *	−10.97%
Ffm, Free fat mass (kg)	56.10 ± 1.95	53.70 ± 1.87	*p* < 0.01 *	−4.23%
Mm, Muscle mass (kg)	53.28 ± 1.85	51.09 ± 1.84	*p* < 0.01 *	−4.16%
Ssm, Skeletal Muscle Mass	31,84 ± 1.09	31.59 ± 0.97	*p* > 0.05	−0.79%
Tbw, Total body water (L)	40.45 ± 1.45	38.39 ± 1.36	*p* < 0.01 *	−5%
Ree, Resting energy expenditure (kcal)	1721.83 ± 56.51	1620.43 ± 52.29	*p* < 0.01 *	−5.77%
RQ, Respiratory quotient	0.94 ± 0.004	0.75 ± 0.004	*p* < 0.01 *	−19.96%
VO_2_ (mL/min)	241.11 ± 47.70	215.16 ± 24.52	*p* > 0.05	−10.76%
VCO_2_ (mL/min)	191.92 ± 52.44	143.58 ± 15.47	*p* > 0.05	−25.18%
VO_2_ (mL/min)/Ffm(kg) Ree/Kcal)/Ffm(kg)	4.41 ± 1.340 30.10 ± 9.61	4.24 ± 0.68 27.98 ± 4.49	*p* > 0.05 *p* > 0.05	−6773% −9474%

The Data are presented as the mean ± standard deviation with * *p* < 0.01.

**Table 3:** Metabolic blood values before and after the revised PSMF diet.

Parameters	Baseline T0 Mean ± SD	T1 Day 21 Mean ± SD	*p*-Value	Δ% T0–T21
Insulin (μU/mL)	20.28 ± 2.57	10.65 ± 0.97	<0.001 *	−36.07%
Glucose (mg/dL)	183.03 ± 4.73	138.43 ± 2.62	<0.001 *	−23.10%
HbA1c (%)	7.98 ± 0.17	7.03 ± 0.16	<0.001 *	−12.04%
HOMA-IR	9.26 ± 6.38	3.65 ± 1.93	<0.001 *	−85.4%
Total cholesterol (mg/dL)	213.1 ± 7.09	169.43 ± 5.47	<0.001 *	−19.74%
HDL cholesterol (mg/dL)	54.1 ± 1.81	51.03 ± 1.55	0.016	−4.51%
LDL cholesterol (mg/dL)	136.93 ± 6.08	103.96 ± 4.43	<0.001 *	−22.48%
Triglycerides (mg/dL)	128.06 ± 6.83	98.4 ± 4.21	<0.001 *	−20.50%
Uric acid (mg/dL)	5 ± 0.15	5 ± 0.21	0.970	0.59%
Ketonemia (mmol/L)	0	1.7 ± 0.06	<0.001 *	

The data are presented as the mean ± standard deviation with * *p* < 0.01.

## Data Availability

The data used to support the findings of this study are available from the corresponding author upon request.
